# Can Population-Level Laterality Stem from Social Pressures? Evidence from Cheek Kissing in Humans

**DOI:** 10.1371/journal.pone.0124477

**Published:** 2015-08-13

**Authors:** Amandine Chapelain, Pauline Pimbert, Lydiane Aube, Océane Perrocheau, Gilles Debunne, Alain Bellido, Catherine Blois-Heulin

**Affiliations:** 1 UMR 6552 Université de Rennes 1—CNRS, Station biologique, 35380, Paimpont, France; 2 UMR 6553 Université de Rennes 1—CNRS, Station biologique, 35380, Paimpont, France; 3 QDC, département R&D, 31000, Toulouse, France; Centre for Ecological and Evolutionary Studies, NETHERLANDS

## Abstract

Despite extensive research, the origins and functions of behavioural laterality remain largely unclear. One of the most striking unresolved issues is the fact that laterality generally occurs at the population-level. Why would the majority of the individuals of a population exhibit the same laterality, while individual-level laterality would yet provide the advantages in terms of improving behavioural efficiency? Are social pressures the key factor? Can social pressures induce alignment of laterality between the individuals of a population? Can the effect of social pressures overpass the effect of other possible determining factors (e.g. genes)? We tested this important new hypothesis in humans, for the first time. We asked whether population-level laterality could stem from social pressures. Namely, we assessed social pressures on laterality in an interactive social behaviour: kissing on the cheek as a greeting. We performed observations in 10 cities of France. The observations took place in spots where people of the city meet and greet each other. We showed that: a) there is a population-level laterality for cheek kissing, with the majority of individuals being aligned in each city, and b) there is a variation between populations, with a laterality that depends on the city. These results were confirmed by our complementary data from questionnaires and internet surveys. These findings show that social pressures are involved in determining laterality. They demonstrate that population-level laterality can stem from social pressures.

## Introduction

Behavioural right/left asymmetries, also called laterality, are widespread in humans and other animals. There is a variety of behavioural asymmetries, including handedness, footedness, eyedness, earness and turning biases. There are two levels of laterality: individual-level laterality is when an individual displays a preference toward one side, population-level or group-level laterality is when the majority of the individuals of a population exhibit the same asymmetry. The most obvious case of population-level laterality is human handedness, with about 90% of individuals being similarly lateralized (i.e. around 90% of right-handers) [1]. In fact, population-level biases are the rule in humans [1], and they have also been frequently observed in other species (see [2, 3, 4, 5, 6, 7, 8, 9] for reviews). Behavioural laterality has long been known and studied, but despite extensive research, many evolutionary and functional issues remain unsolved. The most striking one relates to population-level laterality. Why would the majority of individuals exhibit the same laterality, while individual-level laterality would yet provide the advantages?

Researchers have demonstrated that individual-level laterality would provide advantages [10, 4, 11, 12, 8]. They found empirical evidence showing that laterality would improve the individual behavioural efficiency. Indeed, lateralized individuals have been shown to perform better than unlateralized individuals for a variety of behaviours (in humans, non-human primates and other animals: e.g. [13, 14, 15, 16, 17, 18, 19, 20, 21, 12, 22]. In addition, the direction of laterality may not matter, only the fact that laterality is present is important [10]. This idea is supported by data suggesting that right-sided and left-sided individuals would have similar performances [13, 22].

If laterality provides advantages for the individual performance, whatever its direction, why would the majority of individuals exhibit the same laterality? What causes alignment of the direction of laterality between the individuals of a population? Can this be social pressures? Are social pressures the key to explain population-level biases? In social behaviours, when asymmetrical organisms have to interact with each other, it may be essential for an individual to adapt its laterality according to the asymmetries of the others [23, 24, 6, 8]. For instance, there would be crucial advantages to be lateralized the same way as the other members of the shoal, to avoid being isolated during an escape in front of a predator. There are data in low vertebrates that support this idea (review in [8]). For instance, studies in fishes showed that all the shoaling (i.e. social) species tested exhibited population-level laterality, for the detour task in front of a predator, whereas among the less shoaling (non-social) species, only 40% of the species exhibited population-level laterality [25]. Studies on species with temporary or variable social propensity found that population-level laterality appeared only when the social tendency was present (fishes: [26], tadpoles: [27, 28]). Therefore, there are data suggesting that population-level laterality and social life may be related.

Are social pressures the key to explain population-level biases? Vallortigara and Rogers [8] propose that social pressures may act to align the direction of laterality between the individuals of a population. Their hypothesis concerns survival tasks and involves selection at the genetical level [29].

In the present study, we asked whether social pressures can control laterality, at a non-genetical level. The genetical bases of laterality have been extensively studied and remain matter of research and debate [30, 1, 31]. Genes are generally admitted to have a significant influence on determining laterality, but other factors have been shown to affect laterality, including culture and learning [32, 33, 34, 35, 36]. We are interested in these social aspects. We asked whether social pressures can control laterality. Can social pressures force the individuals to align their asymmetrical behaviour to that of their peers? Can the effect of social pressures overpass the effect of other determining factors (e.g. genes)? This hypothesis is crucial to shed a new light on the evolution and functions of laterality. We tested this hypothesis in humans, for the first time. We asked whether population-level laterality could stem from social pressures. We assessed social pressures on laterality in an interactive social behaviour: cheek kissing.

In France, cheek kissing is the typical greeting act, for saying hello and goodbye. This behaviour is part of the social life and it occurs very frequently. In fact, this is the most common gesture of daily life. The behaviour consists in kissing the other on the cheek, in a sequence of kisses that varies from 1 to 4 kisses per individual, and that starts by the right cheek or the left cheek. The two individuals kiss each other simultaneously. For instance, the kissing individual (A) approaches the kissed individual (B), A kisses B on the left cheek, while B kisses A on the left cheek, then they change cheek to do the next kiss, and so alternate until the end of the kissing sequence. This action involves a contact interaction between the individuals. This interaction is quite complicated to realize. In order to perform the action correctly, the kissing partners must coordinate and adjust their behaviour to each other: they must adjust their posture in order to kiss, they must kiss first on the appropriate cheek, and they must kiss the appropriate number of times. Incorrect or hesitating behaviour causes great embarrassment (pers. observation). For instance, hesitation can result in a “never-ending dance of thrust and withdraw” [37] or to a kiss on the lips. Thus, cheek kissing is considered a nightmare by foreigners (see websites “advices to travellers”). Finally and importantly, in cheek kissing, the partners are numerous and can be unknown persons (even people that meet for the first time may kiss). Therefore, this action is thought to be “a daily miracle of social coordination”. So, it is an ideal model for studying social pressures on a lateralized action.

One must note that cheek kissing is different from lip kissing in couples (kiss of love) that has been studied previously [38, 39, 40, 41, 42]. Firstly, the two behaviours involve different movements: for kissing on the lips in couples, the head is titled to one side and remains in that position for a little while, for kissing on the cheek, the head is tilted to one side for one second to kiss the first kiss and then is titled to the other side to kiss the second kiss and so alternate until the end of the sequence. The kiss is also more precise on the lips compared to the cheek. Secondly, the two behaviours have different value: lip kissing in couples is a sexual behaviour that is directed to one specific partner, cheek kissing is a social behaviour that is directed to numerous persons. Thus, these two behaviours have very different movements, values, emotions, pleasure, meanings and involvments, and they should not be mixed.

When considering the literature on lip kissing in couples, a rightward population-level bias has been consistently reported in western societies [38, 39, 40, 42]. Several hypotheses have been proposed to explain this bias, most hypotheses involve intrinsic factors. The first hypothesis relates to the motor bias in head turning [39]. It proposes that laterality in lip kissing in adults may reflect the persistence of the bias for turning the head to the right that is present in newborns [43, 44, 45, 46, 47, 48, 49]. A second hypothesis proposes that laterality in lip kissing may be related to other lateralities, like handedness, footedness and eyedness [38, 39, 40, 42]. Finally, hypotheses propose that the bias for kissing may be related to brain lateralization for visuomotor control [42] or to brain lateralization for emotions [38]. Indeed, research on baby cradling and posing for portraits suggests that people would act in accordance with their brain lateralization for emotions. People would place themselves so as to expose the more expressive left hemiface to the partner [50, 51, 52] or/and to use the more efficient left visual field to observe the partner [53, 54, 55, 56, 57, 58]. These hypotheses are considered in our study as the intrinsic factor hypothesis. However, we focus on the extrinsic factor hypothesis, related to social pressures.

We propose that laterality for kissing may be controlled by social pressures. Social pressures would force the individuals to align their behaviour to that of their peers within the population. Supporting this idea, the rightward bias for lip kissing that is observed in Western societies is turned into a leftward bias in Middle-East societies [41]. This shows that laterality for kissing can vary between populations, which could suggest that social pressures are involved. However, it is unclear whether this variation is related to social pressures or to reading direction. Indeed, these two populations exhibit opposite reading habits, and left-to-right readers and right-to-left readers are known to differ regarding perceptual and behavioural spatial biases [59], which may affect kissing laterality [41].

In the present study, we examined cheek kissing in France (left-to-right readers). This behaviour is “a daily miracle of social coordination”, so it should be subjected to strong social pressures. We hypothetized that there should be social pressures that force the individuals to adapt to each other, hereby creating alignment between individuals, i.e. population-level laterality. Each individual (even a newly arrived individual) would have to behave so as to fit the others habits. To test this hypothesis, we asked the following questions: Is there population-level laterality for cheek kissing (first cheek kissed)? What are the possible origins of this lateral bias? Can this bias be related to social pressures? We used an ethological approach. We observed cheek kissing in 10 cities of France. We determined which cheek people kiss first in each city. We compared different cities to test whether the behaviour varies between populations, in order to evaluate the influence of social factors. This observational study was complemented by studies using questionnaires and internet surveys that allowed us to extend our investigation. We stated that a) if laterality occurs at the population-level, and b) if laterality varies between different populations, this would show that social pressures are involved.

## Material and Methods

### Ethics

Observations: The observations were anonymous and the data were analyzed anonymously. The observations were done outdoor in public places. We observed the normal daily behaviour. There was no intervention upon people, no interaction with them and no collection of identifiable private data.

Questionnaire: The questionnaire was circulated in 5 universities of France. Moreover, the questionnaire was adapted to internet and was put online. The circulation of the questionnaire was as follows. For the paper version of the questionnaire, we directly gave it to students at the university. For the electronic version of the questionnaire and for the link to the online questionnaire, we sent them via email on diffusion lists of the university (students and staff) and scientific organizations, as well as via social networks. There was no recruitment criterion; everyone could take part in the study, and students gave their oral consent before completing the questionnaire.

### Part 1: observational study

#### Participants and study sites

The observations were carried out in 10 cities of France, in the following order: Montpellier, Toulouse, Aix-en-Provence, Rouen, Rennes, Besançon, Strasbourg, Lyon, Lille and Bordeaux. The observations lasted two weeks in each city. The study was carried out between February 2011 and March 2013. We observed the spontaneous behaviour of people in public places. The subjects were not aware of the study. For the observations, we chose locations that allowed us to optimize data collection, i.e. spots where many people of the city meet and greet each other. The study sites were: entrances of secondary schools in the morning, entrances of university restaurants at lunchtime and meeting spots of the town centre (e.g. the fountain of the main square, entrances of cinemas) in the evening. We only went once to each school, and once or twice to each university restaurant, in order to avoid the risk of recording several times the same individuals. We strived to limit the risk of recording data from people that were foreigners to the city, so we did not use airports and railway stations, and we recorded only data from people that have a typical French type, i.e. white people of western european descent.

#### Data recorded and recording method

The behaviour studied was “kissing on the cheek” as a greeting. It is defined as a kiss on the cheek of the other in a sequence of mutual kissing. We recorded data only when kissing was used as a greeting (to say hello or goodbye), i.e. when the individuals were meeting (or separating, but this case was rarely observed). We used “*ad libitum*” sampling [60] to collect the data, because this is the most appropriate method to record actions that are rapid and infrequent. We scanned the crowd continually and recorded all occurrences of kissing, whenever it occurred. Data collection was made by direct observation with recording on a record sheet.

Definitions: The “initiator” or “kissing individual” is the individual that approaches the other to kiss. The “receiver” is the individual that is kissed first. A “right cheek kiss” occurs when the initiator kisses the receiver on the right cheek first. A “left cheek kiss” occurs when the initiator kisses the receiver on the left cheek first.

For each kissing sequence, we recorded: the first cheek that was kissed, the number of kisses made by the kissing individual (which is the same as the number of kisses made by the individual that is kissed), the sex of the two individuals, the age class of the two individuals (0–10 years, 11–18 years, 18–30 years, 30–50 years, +50 years), the type of dyad (initiator/receiver: Female/Female, Female/Male, Male/Female, Male/Male). This detailed recording was to enable us to investigate and control for the effects of these factors. We also wrote comments about possible influences (e.g. whether the individual held an object that may influence the kissing action, whether the kiss was preceded or followed by a hug) and we recorded whether an individual hesitated, commented or apologized about the kissing action or whether the individuals had problems to kiss correctly. All these data were excluded from the analyses.

#### Data independence

We recorded every instance of kissing. This included repeated data from the same individual. However, these repeated data were used only for the analysis on intra-individual consistency. For the main analysis on kissing laterality, we excluded all cases when an individual was repeated. Thus, if an individual was repeated in several kissing dyads (as the kissing individual or as the kissed individual), we counted only the kiss that occurred in the first dyad. For example, if the kissing individual kissed several persons, we only counted the first instance. If the kissing individual was then the one that was kissed, we only recorded the first instance. These requirements were to ensure that the data analyzed were strictly independent of each others. This resulted in excluding about half of the dataset.

### Part 2: study based on questionnaires and surveys

To complement the observational data, we used questionnaires and surveys.

First, we designed a laterality questionnaire [61] that included 90 items, on manual laterality, podal laterality, visual laterality, auditory laterality and kissing laterality. This last item was the following question: “Imagine that you kiss somebody on the cheek (as a greeting, when we give each other a kiss). Which side of the face would you kiss first? On the drawing, mark with a cross the cheek that you would kiss first (write on or next to the drawing)”. The question was accompanied by the drawing of a face (a circle with two dots for the eyes and a horizontal line for the mouth). This questionnaire has been circulated in 5 universities of France (Aix en Provence, Rennes, Rouen, Strasbourg and Toulouse) between 2010 and 2013. Moreover, this questionnaire was adapted to internet and was put online (since June 2011). The circulation of the questionnaire was as follows. For the paper version of the questionnaire, we directly gave it to students at the university. For the electronic version of the questionnaire and for the link to the online questionnaire, we sent them via email: on diffusion lists of the university (students and staff) and scientific organizations, as well as via social networks. There was no recruitment criterion; everyone could take part in the study. The participants were explained the purpose of the study and asked whether they wanted to take part in it or not. They were told that the data would be analysed anonymously.

**Fig 1:**
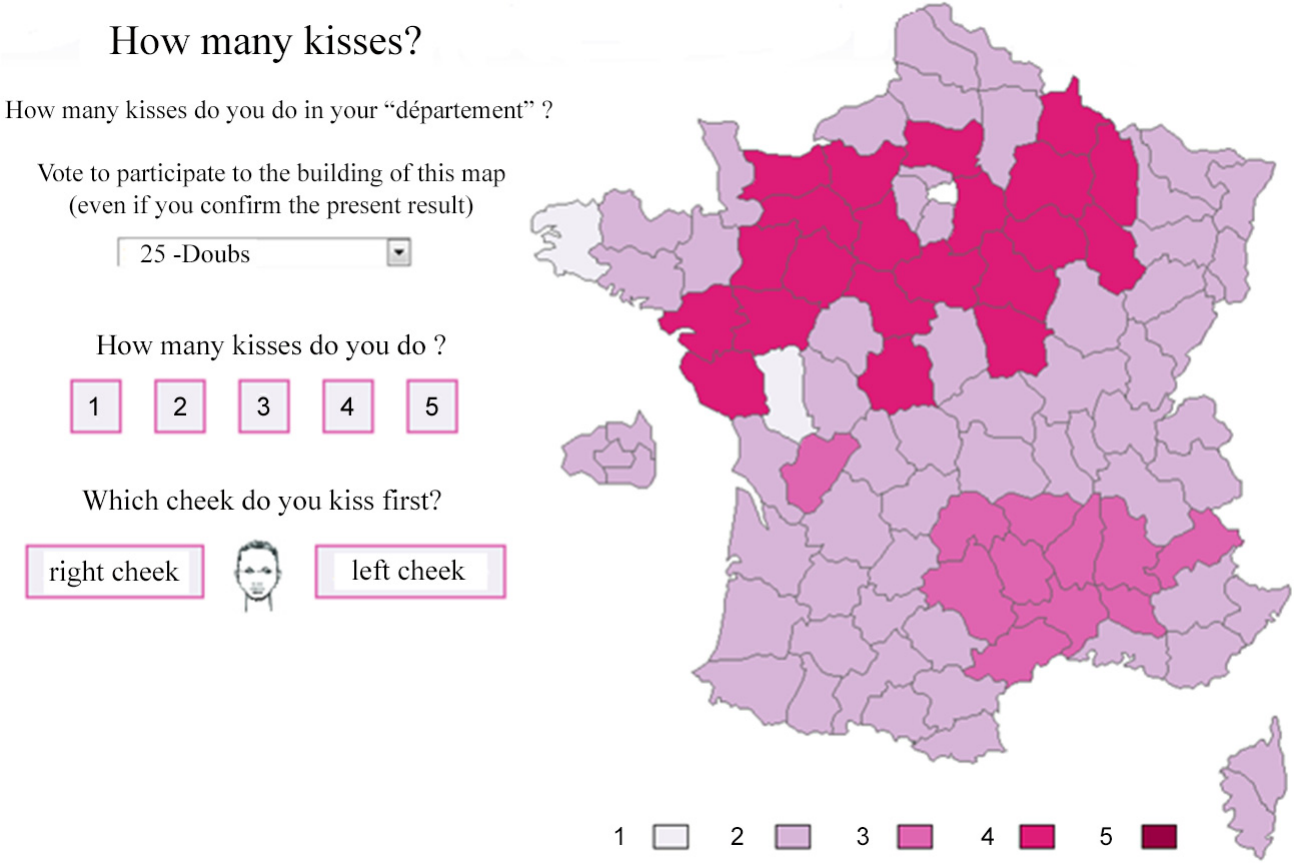
Screenshot of the website “combiendebises” (http://combiendebises.free.fr) (how many kisses).

Second, we used an existing website that is called “combiendebises” (translating as “how many kisses”) (http://combiendebises.free.fr). This website is a running survey (since February 2007). It aims to build a map of France that shows how many kisses are done in each “département” (a “département” is the chief administrative division of France. there are 96 french “départements”). People go on the website and tell how many kisses they do in their “département”. We asked the webmaster, Gilles Debunne, to add the following question: “which cheek do you kiss first?” along with the drawing of a face (Fig 1). This question was added online in April 2011. This internet survey concerned “départements” instead of cities, but we thought that it could provide interesting complementary clues.

### Statistics

We used the Binomial test [62] to compare the number of kisses starting by the left cheek and the right cheek in each city, to determine whether there was a population-level laterality in the city. We also used the commonly used handedness index (HI) to quantify laterality on a continuum. HI was calculated for each city, using the formula: HI = (right–left) / (right + left), where right and left are the numbers of kisses starting by the right cheek and by the left cheek respectively. HI gives the direction of laterality, from -1 to +1, with positive values indicating a bias toward the right cheek and negative values indicating a bias toward the left cheek. The absolute value of HI (absHI) gives the strength of laterality, from 0 to 1.

We investigated the effects of possible influential factors. Regarding laterality, we tested the effects of city, number of kisses, sex and age, using a Generalized Linear Model. Regarding number of kisses, we tested the effects of city, laterality, sex and age, using a Proportional Odds Model. A likelihood ratio test was used to determine the effects of the factors studied. Then multiple comparisons using LSmeans were applied. The following “R” packages were used: ordinal [63], car [64], RVAideMemoire [65] and lsmeans [66].

We examined the geography of laterality using spatial autocorrelation. Spatial autocorrelation occurs when the value of a variable at one locality depends on the values at neighbouring localities [67]. We used Moran's I as spatial autocorrelation coefficient to express the similarities between neighbouring locations [68]. We performed join-counts analysis to test the autocorrelation.

The softwares SPSS, Excel, R, artmap and PASSaGE were used to analyze the data. The statistical tests were considered significant when p ≤ 0.05, “two-tailed”.

## Results

### Part 1: observational study

#### Data analyzed

Sample size: There was a total of 10691 data points, before exclusion of the non-independent data points. We analyzed 5472 independent data points, with an average of 547.20 data points per city (median = 601.50, minimum = 221, maximum = 739).

**Fig 2:**
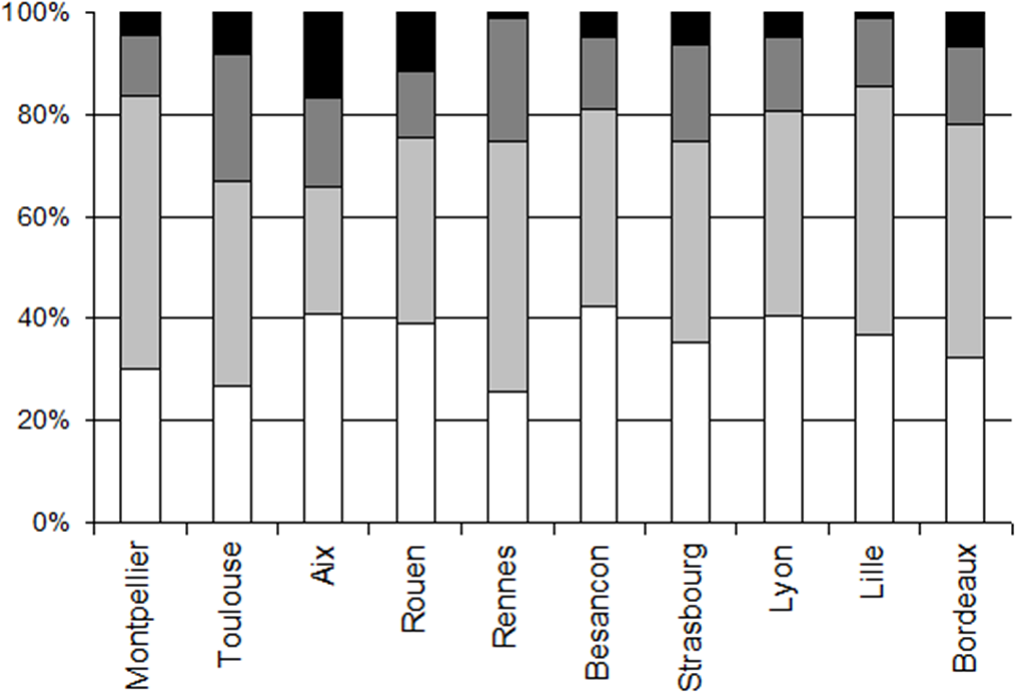
Distribution of the data according to age (kissing individual), in each of the cities. The age classes represented are: 11–18 years (open bar), 18–30 years (light grey), 30–50 years (grey) and +50 years (black).

Distribution of the subjects according to age (Fig 2): In each of the cities, there was virtually no data for the 0–10 years category (less than 2% of the sample of a city), so this age class was excluded from analyses on age. There were few data from the +50 age class (less than 8.2% of the sample of a city), in all the cities, except Rouen and Aix en Provence (11.31% and 16.56% respectively). Therefore, most data points were from the subjects aged between 11 and 30 (at least 65.6% of the data of a city).

**Fig 3:**
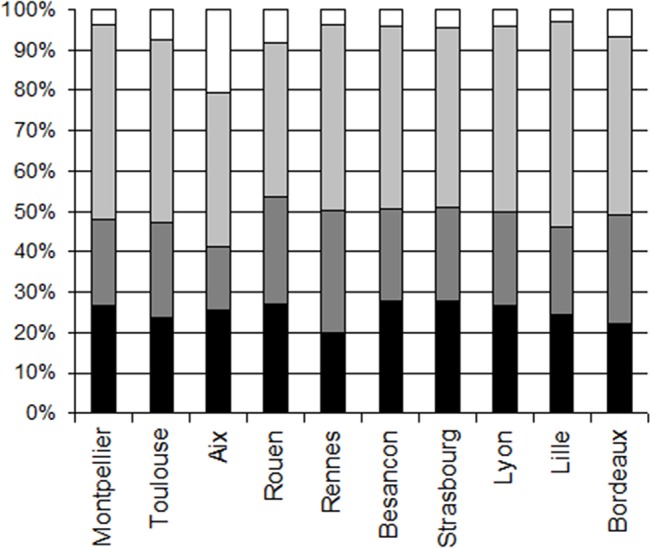
Distribution of the data according to sex, in each of the cities. The dyads are Male/Male (open bar), Female/Female (light grey), Female/Male (grey) and Male/Female (black).

Distribution of the data according to sex (Fig 3): In each of the cities, there were about twice as many data for females compared to males (for both the kissing individual and the kissed individual). When considering the dyads, there were very few data points for Male/Male dyads, in each city (between 3.55% and 8.08% of the data), except in Aix en Provence that exhibited 20.31% of Male/Male dyads.

#### Number of kisses

In each city, the great majority of people (at least 96.5% of the dyads) used the same type of kissing sequence (except Montpellier 71.06%) (Table 1).

**Table 1:** Distribution of the kissing sequences (1 kiss sequences, 2 kisses sequences, 3 kisses sequences, 4 kisses sequences), in each city.

	1 kiss		2 kisses		3 kisses		4 kisses	sum	% majority type
	freq	adj prob	freq	adj prob	freq	adj prob	freq		
Montpellier	143	0.001	35	0.432	**442**	0.567	2	622	71.06
Toulouse	4	0.032	**728**	0.937	5	0.031	2	739	98.51
Aix en Provence	12	0.034	**616**	0.937	7	0.029	3	638	96.55
Rouen	9	0.036	**547**	0.937	1	0.027	0	557	98.21
Rennes	9	0.033	**434**	0.937	0	0.03	0	443	97.97
Besançon	8	0.03	**609**	0.937	3	0.033	2	622	97.91
Strasbourg	5	0.033	**417**	0.937	2	0.03	1	425	98.12
Lyon	5	0.029	**587**	0.937	5	0.034	1	598	98.16
Lille	2	0.028	**219**	0.937	0	0.035	0	221	99.10
Bordeaux	5	0.028	**599**	0.937	1	0.035	0	605	99.01
sum	202		4791		466		11		

Freq: frequency of the kissing sequences (majority types are in bold). Adj prob: adjusted probability of the kissing sequences, based on the likelihood-ratio test and lsmeans. There are no adjusted probabilities for “4 kisses” sequences because this category has been excluded from analysis due to small sample. Sum: total number of data points. % majority type: percentage of people displaying the majority type behaviour.

Effect of the city (Table 1): The number of kisses in a kissing sequence clearly depended on the city. In Toulouse, Aix en Provence, Rouen, Rennes, Besançon, Strasbourg, Lyon, Lille and Bordeaux, the great majority of people (more than 96.5% of the dyads) kissed two times. In Montpellier, the majority of people kissed three times (71.06% of the dyads).

Test of the effects of the city and other factors: We performed analyses using a Proportional Odds Model to determine which factors may influence the number of kisses. We examined the qualitative ordinal variable “number of kisses” and tested the influence of the following factors: city, first cheek kissed, sex of kissing individual, sex of kissed individual, age of kissing individual, age of kissed individual (interactions could not be tested due to small samples for certain categories). For this analysis, categories with very small number of data points were excluded (4 kisses (N = 11), 0–10 years for kissing individual (N = 5), 0–10 years for kissed individual (N = 32)). The model was valid (cond H = 1.2e+03). The likelihood-ratio test shows a significant effect of the city (p<0.001) and laterality (p = 0.032), but no effect of the other factors studied (p≥0.098). Multiple comparisons show significant differences in lsmeans between the cities that enable us to separate Montpellier from the other cities. Table 1 presents the adjusted probabilities of each type of kissing sequences, in each city (Table 1). Regarding the effect of laterality, the adjusted probabilities are the following: 1 kiss: 0.025 for right, 0.019 for left; 2 kisses: 0.936 for right, 0.929 for left, 3 kisses: 0.039 for right, 0.052 for left. Interestingly, when we performed the same analysis without Montpellier, we found no effect of the city (p = 0.57) and no effect of laterality (p = 0.534).

**Fig 4:**
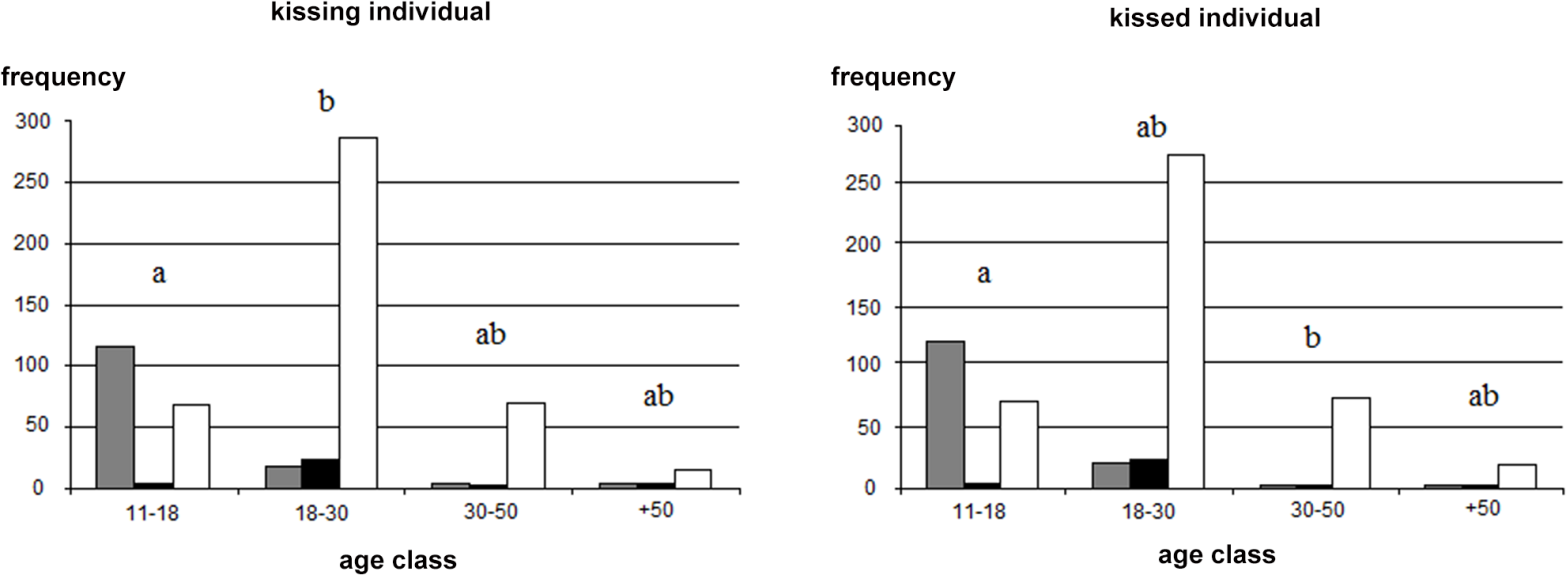
Frequency of the kissing sequences according to age, in Montpellier. The kissing sequences are: 1 kiss sequences (grey), 2 kisses sequences (black), 3 kisses sequences (open bar). Letters represent the differences between age classes, based on the Proportional Odds Model analysis: different letters between two classes indicate a significant difference, similar letters indicate no difference.

Effects of possible influential factors, in each city:—Montpellier: In Montpellier, the majority of people (71.06% of the dyads) kissed three times, but many people (22.99%) kissed one time. When examining the data according to age, we found that there was a higher proportion of “1 kiss” sequences in the 11–18 age class (61%) compared to the other age classes (7.5% on average) (Fig 4). In fact, 61% (61.5% for kissing individual, 60.7% for kissed individual) of the young people (11–18 years old) kissed one time, while 87% (86.9% for kissing individual, 87.2% for kissed individual) of the older people kissed three times (Fig 4). Actually, 82% (81.6% for kissing individual, 82.3% for kissed individual) of the “1 kiss” sequences were performed by subjects of the 11–18 age class. This indicates that the “1 kiss” behaviour would be specific to young people.

As done above, we performed analyses using a Proportional Odds Model on Montpellier only. The model was valid (cond H = 1.5e+03). The likelihood-ratio test shows a significant effect of the age of kissing individual (p = 0.03), the age of kissed individual (p = 0.017), the laterality (p = 0.019), the sex of kissing individual (p = 0.003), but no effect of the sex of kissed individual (p>0.05). Table 2 presents the frequency and the adjusted probabilities of each type of kissing sequences, for each level of the factors (Table 2). Regarding age effects, which is the most marked effect, multiple comparisons show significant differences in lsmeans between the age classes, which enable us to separate the age classes in 2 groups (Fig 4).

**Table 2:** Frequency of the kissing sequences (1 kiss sequences, 2 kisses sequences, 3 kisses sequences, 4 kisses sequences) according to laterality, sex and age, in Montpellier.

	laterality		sex of kissing individual		age of kissing individual				age of kissed individual			
	right	left	female	male	11–18	18–30	30–50	+50	11–18	18–30	30–50	+50
1 kiss	31 (0.188)	110 (0.11)	111 (0.197)	30 (0.105)	115 (0.382)	18 (0.044)	4 (0.132)	4 (0.161)	116 (0.268)	21 (0.243)	2 (0.034)	2 (0.167)
2 kisses	11 (0.076)	23 (0.051)	26 (0.078)	8 (0.049)	4 (0.107)	24 (0.022)	2 (0.059)	4 (0.068)	5 (0.094)	24 (0.089)	2 (0.018)	3 (0.07)
3 kisses	62 (0.736)	376 (0.839)	288 (0.725)	150 (0.846)	68 (0.511)	285 (0.934)	69 (0.809)	16 (0.771)	70 (0.638)	276 (0.668)	73 (0.948)	19 (0.763)
sum	104	509	425	188	187	327	75	24	191	321	77	24

The adjusted probabilities of the kissing sequences, based on the likelihood-ratio test and lsmeans, are given in brackets.

Regarding sex effects, there was a higher proportion of “1 kiss” sequences in females (26.1%) compared to males (16%) (kissing individual). Regarding laterality effects, there was a higher proportion of “3 kisses” sequences in right (59.6%) compared to left (73.9%). However, for these two last effects, there are fewer data in the second category (laterality: five times more data for left, sex: 2.26 times more data for females), which may influence the results.

Other cities: The effects of possible influential factors could not be tested in the other cities because of the small sample size of the minority type (less than 2% of the data of a city). However, given that more than 96.5% of the dyads did the same kissing sequence, we can assume that these factors did not influence the number of kisses.

#### Laterality

In each of the cities, we observed a significant group-level bias (B test p<0.01): the great majority of the individuals of a city (mean = 85.57% of the individuals) started by the same cheek.

**Fig 5:**
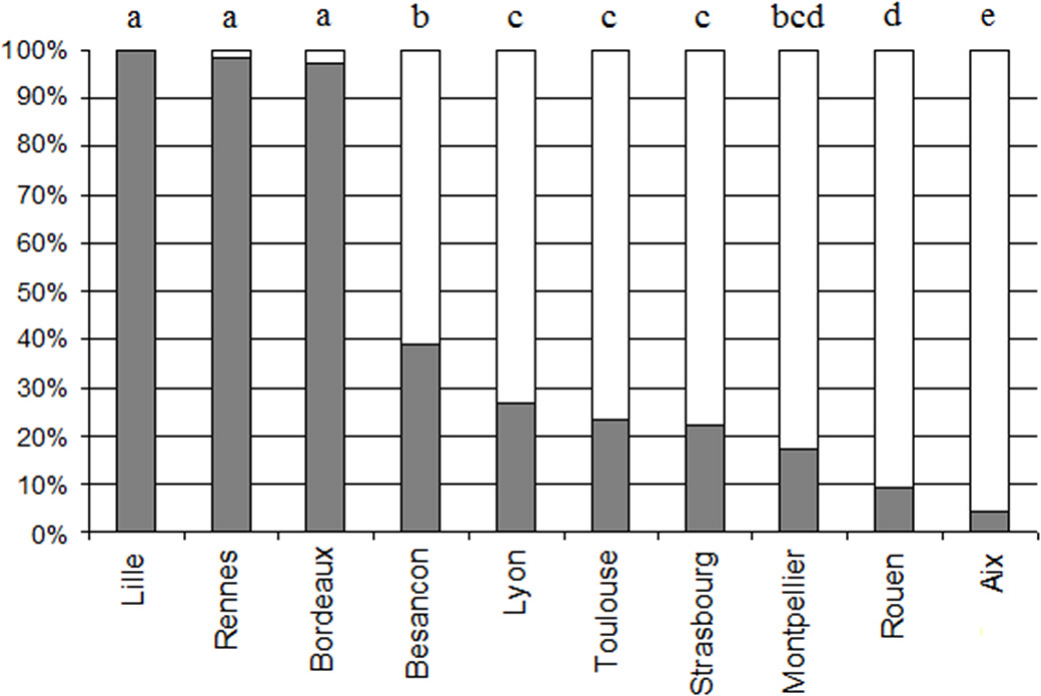
Percentage of kissing sequences starting by the right cheek (grey) and the left cheek (open bar), in each of the cities. Letters represent the differences between cities, based on the Generalized Linear Model analysis: different letters between two cities indicate a significant difference, similar letters indicate no difference.

Effect of the city: The direction of the population-level bias clearly depended on the city: 7 cities were lateralized to the left and 3 cities were lateralized to the right (Fig 5). In Montpellier, Toulouse, Aix en Provence, Rouen, Besançon, Strasbourg and Lyon, the majority of people started to kiss on the left cheek: the number of kisses starting by the left cheek was significantly greater than the number of kisses starting by the right cheek (B test p<0.01) (Table 3). In Rennes, Lille and Bordeaux, the majority of people started to kiss on the right cheek: the number of kisses starting by the right cheek was significantly greater than the number of kisses starting by the left cheek (B test p<0.01).

**Table 3:** Number of kissing sequences starting by the right cheek and the left cheek, in each city.

	right cheek	left cheek	sum	B test	HI	absHI	lateral bias	% majority type	adj prob
Montpellier	107	**515**	622	p<0.01	-0,656	0,656	left bias	82.80	0.9
Toulouse	174	**565**	739	p<0.01	-0.529	0.529	left bias	76.46	0.884
Aix en Provence	28	**612**	640	p<0.01	-0.913	0.913	left bias	95.63	0.981
Rouen	53	**504**	557	p<0.01	-0.810	0.810	left bias	90.49	0.957
Rennes	**437**	6	443	p<0.01	0.973	0.973	right bias	98.65	0.036
Besançon	241	**381**	622	p<0.01	-0.225	0.225	left bias	61.25	0.784
Strasbourg	95	**330**	425	p<0.01	-0.553	0.553	left bias	77.65	0.892
Lyon	160	**438**	598	p<0.01	-0.465	0.465	left bias	75.31	0.86
Lille	**221**	0	221	p<0.01	1	1	right bias	100	5.31e-08
Bordeaux	**590**	15	605	p<0.01	0.95	0.95	right bias	97.52	0.054
sum	2106	3366	5472						

Right cheek: number of kissing sequences starting by the right cheek. Left cheek: number of kissing sequences starting by the left cheek. Majority types are in bold. Sum: total number of data points. B test: p value of the Binomial test performed on the number of right versus left. HI and absHI values. Lateral bias: group-level bias, based on the B test. % majority type: percentage of people displaying the majority type behaviour. Adj prob: adjusted probability for kissing the left cheek, based on the likelihood-ratio test and lsmeans.

The strength of the lateral bias was strong (mean percentage of the majority type = 85.57, median = 86.64, mean absHI = 0.707). In fact, the lateral bias was stronger than 75.3% in all the cities, except Besançon.

Test of the effects of the city and other factors: We performed a Generalized Linear Model analysis to determine which factors may influence the side of first kiss. We examined the variable “first cheek” and tested the influence of the following factors: city, number of kisses, sex of kissing individual, sex of kissed individual, interaction between sex of kissing individual and kissed individual, age of kissing individual, age of kissed individual, interaction between age of kissing individual and kissed individual. For this analysis, categories with very small number of data points were excluded (4 kisses (N = 11), 0–10 years for kissing individual (N = 5), 0–10 years for kissed individual (N = 32)). The validity of the model was confirmed by the equivariance, independence and normality of the residuals. The likelihood-ratio test shows a significant effect of the city (p<0.001) and a trend regarding the number of kisses (p = 0.053), but no effect of the other factors studied (p≥0.203). Multiple comparisons show significant differences in lsmeans between the cities that enable us to group the cities in 5 groups (Fig 5). Table 3 presents the frequency and the adjusted probabilities of each side, for each city (Table 3).

Effects of age and sex: The Generalized Linear Model analysis showed no significant effect of sex and age and interactions (p≥0.203). To confirm this result, we asked whether the population-level bias observed in a city was present in each represented age class (11–18 years, 18–30 years, 30–50 years, +50 years), in each sex category (male and female for the kissing individual, male and female for the kissed individual) and in each type of dyad (Female/Female, Female/Male, Male/Female, Male/Male). We tested only the categories for which the sample size allowed reliable testing, i.e. categories with more than 20 kisses (the age class +50 years could not be tested in Rennes and Lille, the dyads Male/Male could not be tested in Strasbourg and Lille). We found that, in Montpellier, Toulouse, Aix en Provence, Rouen, Rennes, Strasbourg, Lille and Bordeaux, the population-level bias was present in each of the tested categories (Binomial test p<0.05). One can note a sex or age effect in two of the cities. Regarding sex, in Besançon and Lyon, the population-level bias was not observed in the Male/Male dyads (B test p>0.10). However, there were few kisses for this type of dyads (N = 26 and N = 25 respectively), and significant biases are more difficult to reveal in small samples [69]. Regarding age, in Besançon, the population-level bias was not observed in the 11–18 age class (for the kissing individual and for the kissed individual) (B test p>0.53). Thus, the results show no significant and consistent effect of sex or age on laterality.

Effect of the number of kisses:-Montpellier: In Montpellier, we asked whether the population-level bias observed was present in each type of kissing sequences. The bias occurred when people kissed one time (31 right cheek versus 112 left cheek, B test p<0.01) or three times (63 right cheek versus 379 left cheek, B test p<0.01). The bias did not appear when they kissed twice (12 right cheek versus 23 left cheek, B test p = 0.089), but there were few data for 2 kisses sequences (N = 35). As seen above, the Proportional Odds Model analysis showed an effect of laterality on the number of kisses. There was a higher proportion of “3 kisses” sequences in right (59.6%) compared to left (73.9%). However, one must note that there are five times more data for left compared to right, which may influence the results.

- Other cities: In the other cities, the relationship between laterality and number of kisses could not be tested because of the small sample size for the minority types (N ≤ 2% of the data).

Consistency in kissing (Table 4): We assessed intra-individual consistency in kissing, to examine whether the individuals kept their preference across different partners.

**Table 4:** Consistency in kissing (all cities combined).

		over 2 partners	over 3 partners	over 4 partners	over 5 partners	over 6 partners	over 7 partners
kissing individual	number of consistent kissing sequences	1363	468	146	23	13	3
	number of inconsistent kissing sequences	223	101	39	7	4	3
	percentage of consistency	85.94%	82.25%	78.92%	76.67%		
kissed individual	number of consistent kissing sequences	762	106	18	5	1	0
	number of inconsistent kissing sequences	113	24	7	4	0	0
	percentage of consistency	87.09%	81.54%	72%			

The table gives the number of consistent versus inconsistent kissing sequences observed. We separated cases when an individual kisses several persons (line: kissing individual) and cases when an individual is kissed by several persons (line: kissed individual). The columns give the number of partners that interact with the individual. ex: there were 468 cases when a kissing individual was consistent in kissing three different partners, the percentage of consistency is the proportion of consistent kissing sequences over the total occurences of 3 partners kisses.

We first assessed the consistency of the kissing individuals (in each city and all cities combined). In each city, we found that most individuals were consistent, over 2 partners (mean = 87.42%) or 3 partners (mean = 84.63%). The sample of data did not allow analysis on 4 partners and more. One should note that there were cases of consistency over up to 7 different partners. These findings show that most individuals were consistent, kissing all of their partners starting by the same cheek.

We also examined the consistency within the kissed individuals (in each city and all cities combined). In each city, we found consistency in most cases, over 2 partners (mean = 87.94%). The sample of data did not allow analysis on 3 partners and more. One should note that there were cases of consistency over up to 6 different partners. This shows that there was a high consistency in the kissing side regarding the kissed individuals.

### Part 2: study based on questionnaires and surveys

#### Questionnaires

Paper version questionnaire—cheek kissing: We had data from two cities for which we have also performed behavioural observations: Rennes and Rouen. The questionnaires revealed significant population-level biases in both cities: a right bias in Rennes (60 right kisses versus 9 left kisses, B test p<0.01, 86.96% majority type) and a left bias in Rouen (26 right kisses versus 78 left kisses, B test p<0.01, 75% majority type).

Online version questionnaire—cheek kissing (Table 5): We obtained 2815 responses for the online questionnaire. We analyzed only cities for which there was a minimum of 20 responses. The results show significant population-level laterality in 7 cities (and one trend). The biases were all toward the right cheek. The 7 other cities analyzed showed no significant bias.

**Table 5:** Number of responses for kissing sequences starting by the right cheek and the left cheek, in each city (results of the online questionnaire).

	right cheek	left cheek	sum	B test	lateral bias	% majority type
Angers	23	19	42	p = 0.644	no bias	
Besancon	22	23	45	p = 1	no bias	
Brest	25	7	32	p = 0.002	right bias	78.13
Laval	21	4	25	p = 0.001	right bias	84
Le Mans	28	13	41	p = 0.028	right bias	68.29
Lille	19	9	28	p = 0.087	no bias	
Nantes	26	13	39	p = 0.053	right trend	66.67
Paris	48	17	65	p = 0.0001	right bias	73.85
Quimper	27	2	29	p<0.0001	right bias	93.1
Rennes	174	80	254	p<0.0001	right bias	68.5
Saint brieuc	23	12	35	p = 0.09	no bias	
Saint malo	15	12	27	p = 0.701	no bias	
Strasbourg	11	11	22	p = 1	no bias	
Toulouse	7	14	21	p = 0.189	no bias	
Vannes	22	9	31	p = 0.029	right bias	70.97

see legend for Table 3.

Online version questionnaire—cheek kissing and handedness: For the online questionnaire, we had data on both handedness and cheek kissing, so we could investigate the relationship between the two lateralities. When considering the writing hand, there were 1846 right-handers and 278 left-handers. The percentage of kisses starting by the left cheek was 35.97% in right-handers and 38.85% in left-handers, showing no effect of handedness on laterality for cheek kissing (B test p>0.05).

#### Internet website “combiendebises”

**Fig 6:**
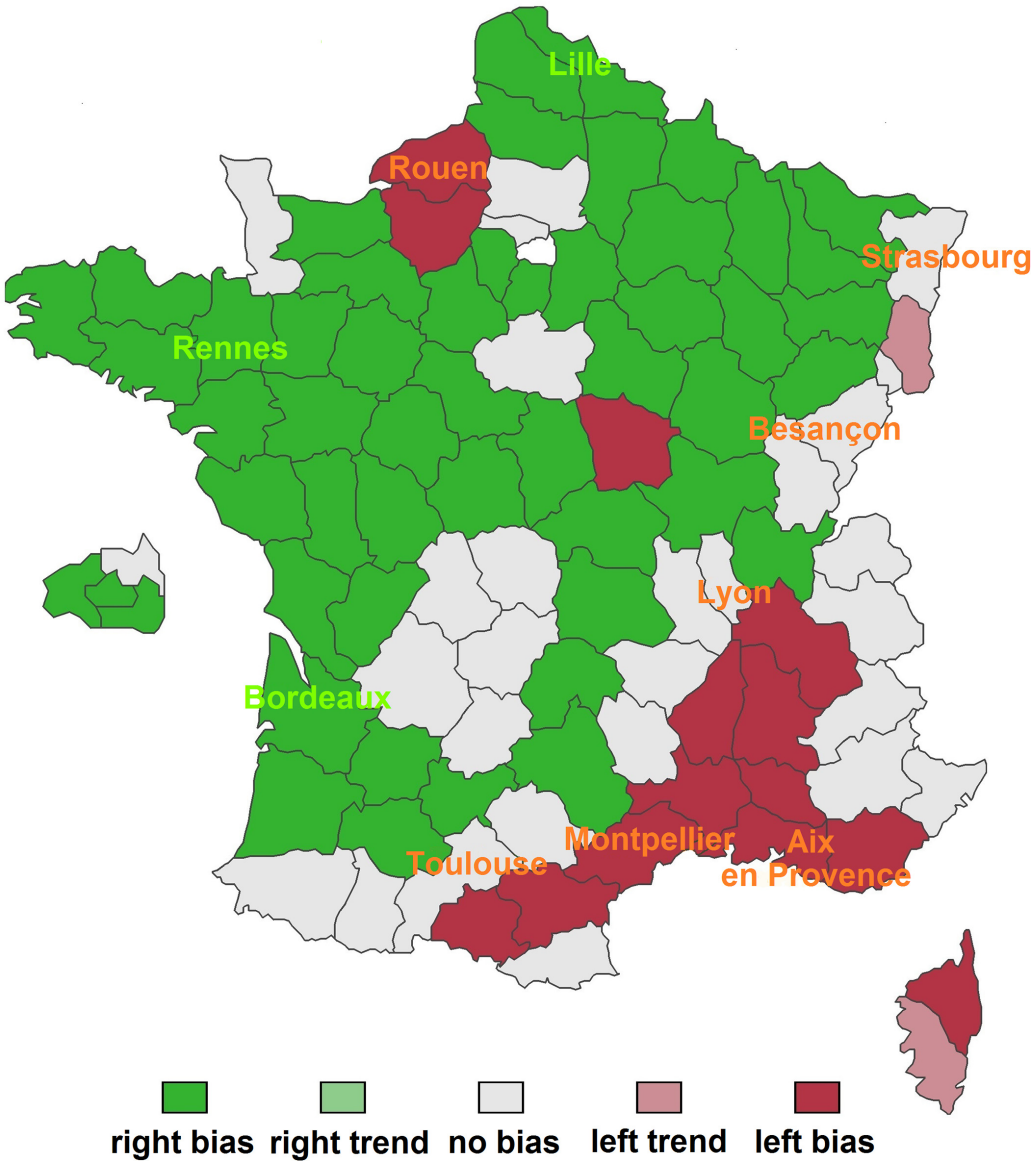
Map presenting the results of the direct observations and the results of the website “combiendebises” (http://combiendebises.free.fr) (how many kisses). City names show the results of the direct observations, which are based on cities. Green represents right biases and red represents left biases. Background colours show the results of the internet website, which are based on “départements”.

We obtained 43200 responses for the online survey on the 96 “départements” of France. There were 450 responses per “département” on average (median = 327, minimum = 57, maximum = 4017). There was no correlation between the number of data points and the strength of laterality (Spearman rho = 0.092, p = 0.373, N = 96). The analyses show significant population-level laterality in 66 “départements” (Table 6, Fig 6). There were 52 “départements” lateralized toward the right cheek (B test p≤0.05). There were 14 “départements” lateralized toward the left cheek (B test p≤0.05). There were 2 “départements” showing a non-significant trend toward the left cheek (B test p≤0.075). The 28 other “départements” showed no trend or bias (B test p>0.05).

**Table 6:** Number of responses for kissing sequences starting by the right cheek and the left cheek, in each “département” (results of the website “combiendebises”).

"département"	right cheek	left cheek	sum	B test	lateral bias	% majority type
Ardeche	136	253	389	p<0.0001	left	65.04
Ariege	35	56	91	0.035	left	61.54
Aude	116	151	267	0.037	left	56.55
Bouches du Rhone	519	706	1225	p<0.0001	left	57.63
Drome	248	377	625	p<0.0001	left	60.32
Eure	136	191	327	0.003	left	58.41
Gard	262	312	574	0.041	left	54.36
Haute Corse	29	47	76	0.050	left	61.84
Herault	631	751	1382	0.001	left	54.34
Isere	323	479	802	p<0.0001	left	59.73
Nievre	72	102	174	0.028	left	58.62
Seine Maritime	225	294	519	0.003	left	56.65
Var	153	220	373	0.001	left	58.98
Vaucluse	198	309	507	p<0.0001	left	60.95
Corse du Sud	40	60	100	0.057	left trend	60.00
Haut Rhin	135	167	302	0.074	left trend	55.30
Ain	390	254	644	p<0.0001	right	60.56
Aisne	172	96	268	p<0.0001	right	64.18
Allier	146	76	222	p<0.0001	right	65.77
Ardennes	131	85	216	0.002	right	60.65
Aube	111	72	183	0.005	right	60.66
Aveyron	165	110	275	0.001	right	60.00
Calvados	396	216	612	p<0.0001	right	64.71
Cantal	92	51	143	0.001	right	64.34
Charente	200	85	285	p<0.0001	right	70.18
Charente Maritime	309	112	421	p<0.0001	right	73.40
Cher	129	70	199	p<0.0001	right	64.82
Cote d'Or	216	123	339	p<0.0001	right	63.72
Cotes d'Armor	367	137	504	p<0.0001	right	72.82
Deux Sevres	299	98	397	p<0.0001	right	75.31
Essonne	332	179	511	p<0.0001	right	64.97
Eure et Loir	138	69	207	p<0.0001	right	66.67
Finistere	1142	308	1450	0	right	78.76
Gers	59	35	94	0.017	right	62.77
Gironde	583	250	833	p<0.0001	right	69.99
Haute Marne	81	41	122	0.0004	right	66.39
Haute Saone	52	33	85	0.050	right	61.18
Hauts de Seine	521	242	763	p<0.0001	right	68.28
Ille et Vilaine	770	268	1038	0	right	74.18
Indre	77	47	124	0.009	right	62.10
Indre et Loire	300	246	546	0.023	right	54.95
Landes	109	61	170	0.0003	right	64.12
Loir et Cher	145	68	213	p<0.0001	right	68.08
Loire Atlantique	889	397	1286	0	right	69.13
Lot et Garonne	80	37	117	p<0.0001	right	68.38
Maine et Loire	418	201	619	p<0.0001	right	67.53
Marne	226	92	318	p<0.0001	right	71.07
Mayenne	144	61	205	p<0.0001	right	70.24
Meurthe et Moselle	322	129	451	p<0.0001	right	71.40
Meuse	82	48	130	0.004	right	63.08
Morbihan	472	166	638	p<0.0001	right	73.98
Moselle	338	148	486	p<0.0001	right	69.55
Nord	849	356	1205	0	right	70.46
Orne	140	78	218	p<0.0001	right	64.22
Paris	2681	1336	4017	0	right	66.74
Pas de Calais	324	173	497	p<0.0001	right	65.19
Puy de Dome	273	107	380	p<0.0001	right	71.84
Saone et Loire	159	59	218	p<0.0001	right	72.94
Sarthe	224	133	357	p<0.0001	right	62.75
Seine et Marne	375	244	619	p<0.0001	right	60.58
Somme	130	81	211	0.001	right	61.61
Tarn et Garonne	59	32	91	0.006	right	64.84
Val de Marne	386	169	555	p<0.0001	right	69.55
Vendee	334	115	449	p<0.0001	right	74.39
Vienne	252	83	335	p<0.0001	right	75.22
Vosges	138	62	200	p<0.0001	right	69.00
Yonne	103	67	170	0.007	right	60.59
Yvelines	459	220	679	p<0.0001	right	67.60

see legend for Table 3. Only the “départements” that display a significant bias are represented. All the other “départements” have similar numbers of right and left responses.

### Part 3: Consistency between the different methods

The results based on the questionnaires and survey were concordant with the findings based on the direct observations (Fig 6). That is, when significant biases were revealed by the different methods, they were in the same direction. A few inconsistencies can be noted, but this was regarding unlateralized versus lateralized places, not regarding direction of laterality. Thus, these differences were related to the strength of laterality. When we examined the strength of laterality in the different methods, differences appeared. The laterality tended to be stronger in the observational study compared to the questionnaires and survey. For instance, in Rennes, the right bias was 98.65% with the direct observations, 86.96% with the paper version questionnaire, 68.5% with the online questionnaire and the bias for the “département” of Rennes was 74.18% with the survey.

### Part 4: Geography / spatial distribution

**Fig 7:**
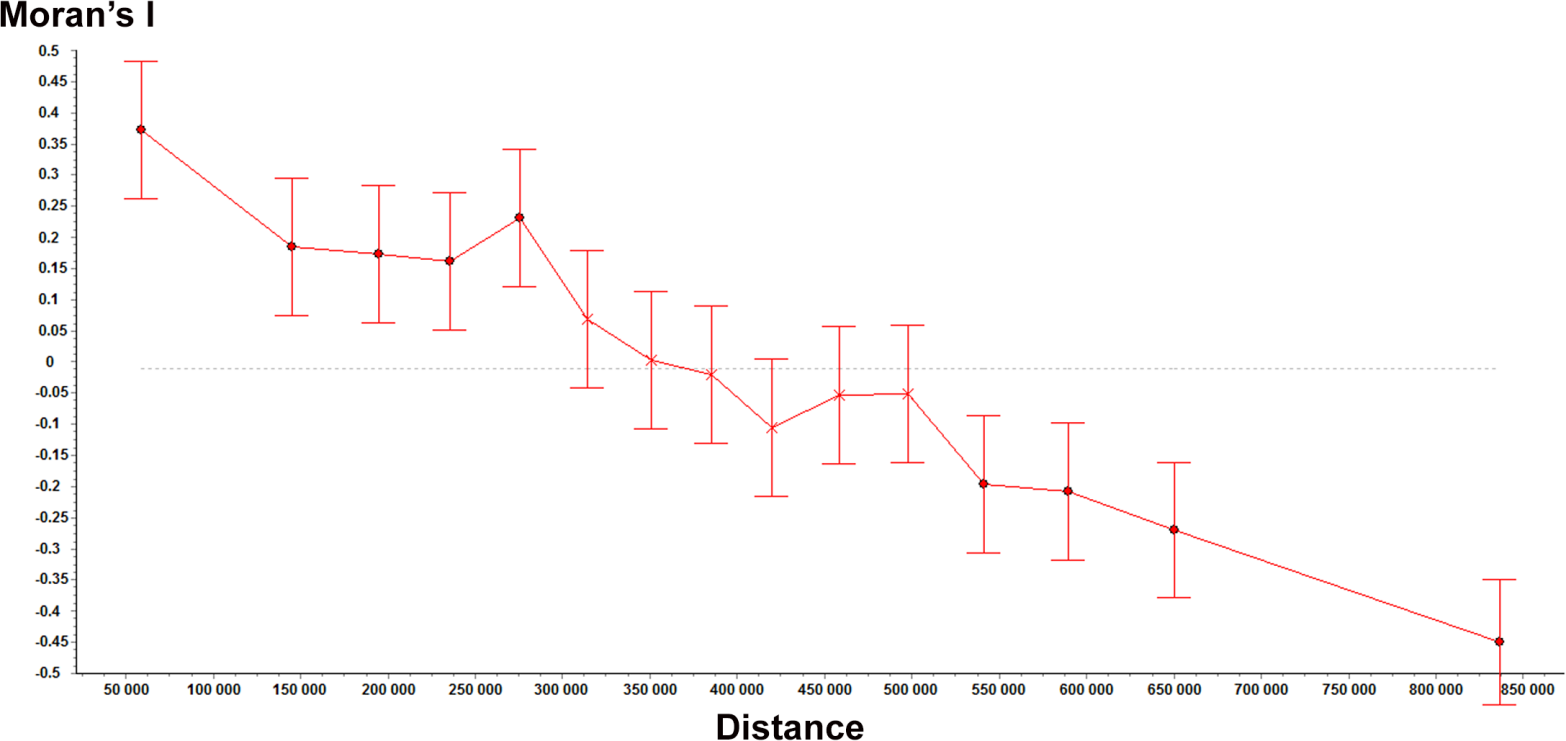
Correlogram showing the Moran’s intercorrelation index (I) according to distance. This analysis is based on the HI values of 94 “départements”.

**Fig 8:**
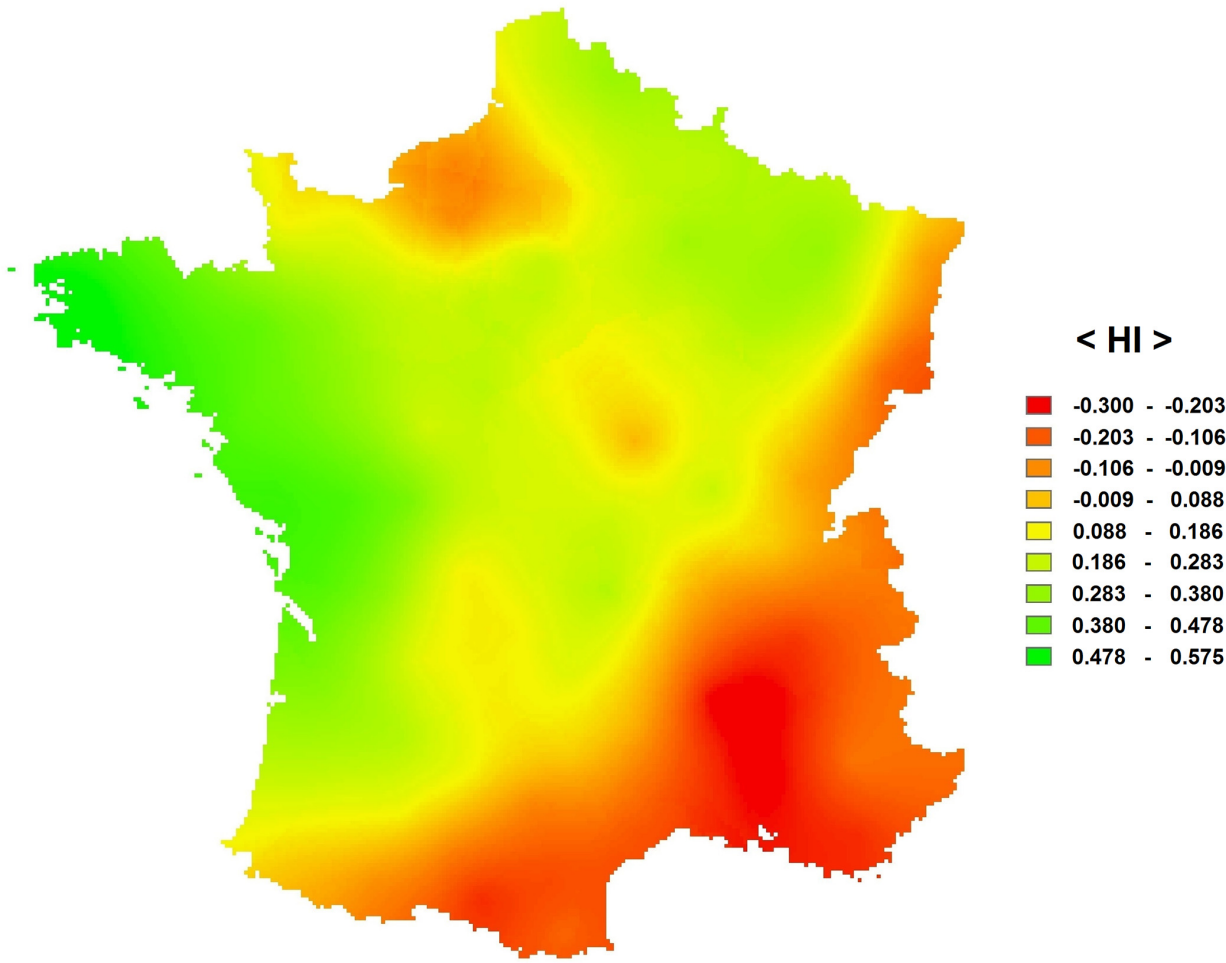
Map built using ordinary kriging interpolation method. This analysis is based on the HI values of 94 “départements”.

We examined the spatial distribution of right-sided and left-sided populations. This analysis was based on the internet survey for the “départements”. Fig 6 presents the geographical location of the “départements” classified in 5 categories based on the B test (left trend, left bias, no bias, right trend, right bias) (Fig 6, Table 6). We performed a spatial analysis that included 94 “départements”. Two “départements” (Haute Corse, Corse du Sud) were excluded because they were located on an island so were isolated from the other “départements”. The spatial coordinates of the “départements” were those of the centroids of the “départements”. We grouped the data into 15 distance classes of equal number of observations. The Gabriel network [70] was used as connection basis between the data points. With this network, any two localities A and B are considered connected when no other locality lies on or within the circle of diameter A-B. The join-counts analysis with permutation test shows a significant correlation between “left” x “left” (p<0.001) and between “right” x “right” (p<0.001), but no correlation between “NS” x “NS” (p = 0.23). This result means that the “départements” that are close neighbours (based on the Gabriel network connections) are likely to show similar patterns of laterality (when they are lateralized). Not lateralized “départements” were not spatially correlated. We built a correlogram based on the HI values. The correlogram also shows a correlation between the data points that are geographically close (Fig 7). Namely, there is a significant positive correlation for distance classes 1, 2, 3, 4 and 5 (distances 0–296962 km) (Moran, p<0.001) and a negative correlation for distance classes 12, 13, 14 and 15 (distances 518194–987394 km) (Moran, p<0.001). Therefore, the analyses reveal a spatial structure of laterality. Fig 8 presents a map built using ordinary kriging interpolation method, based on HI values (Fig 8). It shows a North-East—South-West diagonal, with most right-sided populations being located above this diagonal and most left-sided populations being located below this diagonal.

## Discussion

One of the most striking unresolved issues regarding laterality is the origin of group-level biases. Can social pressures be the key factor that induces alignment of laterality between the individuals? We tested this hypothesis in humans, for the first time. Namely, we asked whether social pressures could control laterality for an interactive social behaviour. We studied laterality for cheek kissing, a behaviour that involves a contact interaction with numerous partners. Our observations in 10 French cities showed that a) the majority of the people of a city performed the same action and b) the direction of laterality depended on the city. These results are consistent with our complementary data for questionnaires and surveys.

### Number of kisses

In each city, the majority of people used the same kissing sequence. In Toulouse, Aix en Provence, Rouen, Rennes, Besançon, Strasbourg and Lyon, the majority of people kissed two times. In Montpellier, the majority of people kissed three times, but many people kissed one time. When examining the data according to age, we found that most of the young people (11–18 years old) kissed one time, while the older people kissed three times. Thus, there were two populations in Montpellier: people under 18 who kissed one time and people over 18 who kissed three times. Thus, our results show that the number of kisses depended on the city and on the age class. In each population—based on city and age class–the majority of people used the same kissing sequence. Thus, each population exhibited a specific kissing sequence that was used by the majority of people. This finding shows that the people of a population would be subjected to social pressures that force them to adapt their behaviour to that of their peers.

Our study is the first observational study on cheek kissing. Our results are consistent with the data from the internet survey (http://combiendebises.free.fr/), indicating that the number of kisses would vary depending on the “département”. Together the data show that the behavioural pattern differs between populations. This suggests that it would be related to social pressures that are specific to each population. So, cheek kissing would be shaped by social pressures regarding the number of kisses.

### Laterality

#### Observational study

In each city, we observed a significant group-level bias: the majority of the individuals of a city started by the same cheek. This result demonstrates that there is a population-level laterality for cheek kissing. The lateral bias was very marked: on average, 85.57% of the people of a city exhibited the same behaviour. One must note that, in Aix en Provence, Bordeaux, Rennes and Lille, there was virtually no people of the minority type (less than 4.4% of the people). Thus, our finding confirms our first hypothesis: laterality occurs at the population-level.

What are the origins of this population-level bias? Is it related to intrinsic factors or to extrinsic factors? Firstly, we consider hypotheses on intrinsic factors. One can propose that the observed group-level laterality may stem from intrinsic factors that are standardized amongst humans. For instance, there may be a genetical basis for laterality biases that influence kissing behaviour. As suggested by Güntürkün [39], kissing behaviour could be influenced by the head turning rightward bias that is present in newborns [44, 49]. Kissing could also be related to brain lateralization for visuomotor control [42] or to brain lateralization for emotions [38]. Finally, kissing could be related to other lateralities, like handedness, footedness and eyedness [38, 39, 40, 42]. Secondly, we consider hypotheses on extrinsic factors. One can propose that the observed group-level bias may stem from social pressures that force alignment between the individuals. In a population, each individual (even a newly arrived individual) would have to behave so as to fit the others habits.

If laterality appears to be consistent between a number of different populations and contexts (i.e. universal), this would suggest that it is related to intrinsic factors. If laterality varies, this would indicate that extrinsic factors are involved. In the present study, we tested whether laterality could be controlled by extrinsic factors: social pressures. We investigated the effect of the place and found that the place is the key determining factor. Indeed, the laterality clearly depended on the place considered: 7 cities exhibited a bias toward the left cheek and 3 cities exhibited a bias toward the right cheek. This result confirms our second hypothesis: laterality varies between populations.

#### Study based on questionnaires and surveys

The findings from the observational study are supported and extended by our second study that is based on questionnaires and surveys. Indeed, we also found population-level laterality and variation of laterality between places in the second study.

Moreover, the results based on the questionnaires and survey were consistent with the findings based on the direct observations. That is, when significant biases were revealed by the different methods, they were in the same direction. One can note that there were a few inconsistencies, but this was regarding unlateralized versus lateralized places, i.e. strength not direction of laterality. Namely, laterality appeared weaker in the internet survey, with a number of “départements” being not lateralized. This could be due to differences in the sample of data. Indeed, the survey is based on “départements” instead of cities. A “département” can include several cities that are not necessarily similarly lateralized, which may create a negative result for the “département”. Supporting this view, the direct observations showed a significant laterality bias in the cities of Strasbourg, Besançon, Lyon and Toulouse, while the internet survey showed no bias for the corresponding “départements”.

One could also wonder whether the data from the questionnaires and survey were perfectly representative of the real pattern, or whether they could have been slightly biased, by a misunderstanding of the question or by a mistake when reporting laterality (see discussions on the reliability of reported compared to actual laterality, e.g. [15]). The good consistency between the data from the questionnaire or survey and the observational data shows that these findings were reliable.

#### Social pressures

Therefore, our results from both studies clearly show that a) there is a population-level laterality for cheek kissing, and b) there is a variation of laterality between populations.

What can explain this variation between populations? Can this be social pressures or other factors? Reading habit cannot be related to kissing laterality here [59] because the studied populations all exhibited left-to-right reading. A genetical variation between populations is highly unlikely because the studied populations were geographically very close to each other. Moreover, laterality biases (handedness, footedness, etc…) are known to be universal features [71, 15, 72] that show limited variations between populations (e.g. right-handers proportion only varies between 73% and 100% (e.g. [1, 73, 74, 75]. Thus, the best explanation to our results is social. Our findings show that laterality would be related to social pressures that vary between populations.

A further supportive point for the social related hypothesis is the fact that the observed laterality was so strong (on average 85.57% of the individuals aligned in a city) (more than 95% in Aix en Provence, Bordeaux, Rennes and Lille), while there was a significant proportion of people that have not lived all their life in that city (e.g. students from other cities). The strong laterality shows that even newly arrived individuals behave in accordance with the population. This means that an individual that comes from a right sided city is forced to change to left side on arrival in a left sided city.

Therefore, our findings demonstrate that an extrinsic factor—social pressures—is the key determining factor that explains laterality for cheek kissing. Social pressures would force the individuals to adapt their behaviour to that of their peers, hereby creating alignment of laterality and population-level bias.

In the literature, there is evidence of socio-cultural effects on laterality. The most obvious exemple concerns handedness. Indeed, some societies exhibit strong cultural pressures against left-handedness, which affects the population left-handers/right-handers ratio [76, 33]. However, in this case socio-cultural effects would be secondary factors. Cultural aspects would influence laterality, but these factors would act along with other determining factors (e.g. genes). Our results overpass previous findings in that they are the first to show that laterality can be controlled by social pressures. Indeed, in our study, social pressures are the key determining factor that overpasses the effects of other possible determining factors.

Another interesting difference concerns the way social pressures act. In cheek kissing, alignment of laterality is made through non-explicit social pressures. There is no explicit rule or education for learning which side to kiss. This is an individual learning through experience in a given population. One can note that alignment of laterality for cheek kissing is not crucial in terms of survival, yet, it occurs.

When we consider handedness, there is an explicit banning on left-hand use and learning occurs during the education of the children. One can think that this education against left hand use may be important in terms of survival. For instance regarding social work with dangerous tools, such as reaping [1]. Moreover, in certain modern societies, with high level of poverty (e.g. african societies), toilet paper and soap are not available. People do not use toilet paper but use their hand and water to wash in the toilet. Moreover, they rarely use soap to wash their hands. In such societies, it would be crucial to separate the roles of the hands: one hand used in the toilet, one hand used for all other activities. Separating the roles of the hands would be important for the survival of the individual. Establishing strong social rules to align the direction of asymmetries between the individuals of the population would be crucial, particularly when considering behaviours like eating with the hand in a shared dish and greeting people with hand shake. One can imagine that this explanation can also apply to ancient human societies. Thus, social pressures may have been an important factor in the evolution of laterality, acting alone or together with other determinants (e.g. genes).

In conclusion, we assessed social pressures on an interactive social behaviour and found that: a) there is a population-level laterality and b) there is a variation of laterality between populations. These results show that social pressures are involved in the determination of laterality for cheek kissing. Here, social pressures would be the key factor, acting to force alignment between the individuals. Therefore, our findings demonstrate that social pressures can induce population-level laterality. This challenges long standing views on the determinants of laterality, suggesting that social pressures can be a particularly important factor, that could overpass other determinants of laterality. Future investigation may study the history of each of the cities, to determine the origins of the direction of laterality in each city.

## Supporting Information

S1 Table These are the raw data of Fig 2. Distribution of the data according to age (kissing individual), in each of the cities.
(DOC)

S2 Table These are the raw data of Fig 3. Distribution of the data according to sex, in each of the cities.
(DOC)

S3 Table These are the raw data of Fig 4. Frequency of the kissing sequences according to age, in Montpellier.
(DOC)

S4 Table These are the raw data of Fig 7. Correlogram showing the Moran’s intercorrelation index (I) according to distance.
(DOC)
