# Correction: Can Population-Level Laterality Stem from Social Pressures? Evidence from Cheek Kissing in Humans

**DOI:** 10.1371/journal.pone.0148456

**Published:** 2016-01-28

**Authors:** Amandine Chapelain, Pauline Pimbert, Lydiane Aube, Océane Perrocheau, Stéphanie Barbu, Gilles Debunne, Alain Bellido, Catherine Blois-Heulin

Dr. Stéphanie Barbu should be included in the author byline. She should be listed as the fifth author, and her affiliation is 4: UMR 6552 University of Rennes 1—CNRS, Campus de Beaulieu, 35040 Rennes Cedex, France.

The correct citation is: Chapelain A, Pimbert P, Aube L, Perrocheau O, Barbu S, Debunne G et al. (2015) Can Population-Level Laterality Stem from Social Pressures? Evidence from Cheek Kissing in Humans. PLoS ONE 10(8): e0124477. doi:10.1371/journal.pone.0124477
