# Supplementary material for: Can Population-Level Laterality Stem from Social Pressures? Evidence from Cheek Kissing in Humans
Source: PLoS One. 2015 Aug 13;10(8):e0124477. doi: 10.1371/journal.pone.0124477 (PMC4536016; doi:10.1371/journal.pone.0124477)
Supplement: S1 Table — Distribution of the data according to age (kissing individual), in each of the cities. (DOC) [file pone.0124477.s001.doc]

**S1 Table. These are the raw data of Figure 2. Distribution of the data according to age (kissing individual), in each of the cities.**

|  | 0-10 years | 11-18 years | 18-30 years | 30-50 years | +50 years | sum |
| --- | --- | --- | --- | --- | --- | --- |
| Montpellier | 1 | 187 | 329 | 76 | 25 | 618 |
| Toulouse | 0 | 196 | 297 | 180 | 60 | 733 |
| Aix | 1 | 262 | 158 | 113 | 106 | 640 |
| Rouen | 1 | 217 | 202 | 74 | 63 | 557 |
| Rennes | 0 | 114 | 218 | 106 | 5 | 443 |
| Besancon | 0 | 263 | 242 | 87 | 30 | 622 |
| Strasbourg | 2 | 150 | 167 | 80 | 26 | 425 |
| Lyon | 0 | 243 | 240 | 86 | 29 | 598 |
| Lille | 0 | 82 | 107 | 30 | 2 | 221 |
| Bordeaux | 0 | 196 | 275 | 92 | 41 | 604 |
| sum | 5 | 1910 | 2235 | 924 | 387 | 5461 |
