# Supplementary material for: Can Population-Level Laterality Stem from Social Pressures? Evidence from Cheek Kissing in Humans
Source: PLoS One. 2015 Aug 13;10(8):e0124477. doi: 10.1371/journal.pone.0124477 (PMC4536016; doi:10.1371/journal.pone.0124477)
Supplement: S2 Table — Distribution of the data according to sex, in each of the cities. (DOC) [file pone.0124477.s002.doc]

**S2 Table. These are the raw data of Figure 3. Distribution of the data according to sex, in each of the cities.**

|  | MF | FM | FF | MM | sum |
| --- | --- | --- | --- | --- | --- |
| Montpellier | 167 | 132 | 300 | 22 | 621 |
| Toulouse | 175 | 173 | 333 | 55 | 736 |
| Aix | 163 | 100 | 247 | 130 | 640 |
| Rouen | 151 | 147 | 214 | 45 | 557 |
| Rennes | 89 | 133 | 205 | 16 | 443 |
| Besancon | 172 | 143 | 281 | 26 | 622 |
| Strasbourg | 119 | 98 | 189 | 19 | 425 |
| Lyon | 161 | 138 | 274 | 25 | 598 |
| Lille | 54 | 48 | 112 | 7 | 221 |
| Bordeaux | 136 | 163 | 266 | 40 | 605 |
| sum | 1387 | 1275 | 2421 | 385 | 5468 |
