# Supplementary material for: Can Population-Level Laterality Stem from Social Pressures? Evidence from Cheek Kissing in Humans
Source: PLoS One. 2015 Aug 13;10(8):e0124477. doi: 10.1371/journal.pone.0124477 (PMC4536016; doi:10.1371/journal.pone.0124477)
Supplement: S3 Table — Frequency of the kissing sequences according to age, in Montpellier. (DOC) [file pone.0124477.s003.doc]

**S3 Table. These are the raw data of Figure 4. Frequency of the kissing sequences according to age, in Montpellier.**

Kissing individual:

|  | 11-18 years | 18-30 years | 30-50 years | +50 years | sum |
| --- | --- | --- | --- | --- | --- |
| 1 kiss | 115 | 18 | 4 | 4 | 141 |
| 2 kisses | 4 | 24 | 2 | 4 | 34 |
| 3 kisses | 68 | 285 | 69 | 16 | 438 |
| sum | 187 | 327 | 75 | 24 | 613 |

Kissed individual:

|  | 11-18 years | 18-30 years | 30-50 years | +50 years | sum |
| --- | --- | --- | --- | --- | --- |
| 1 kiss | 116 | 21 | 2 | 2 | 141 |
| 2 kisses | 5 | 24 | 2 | 3 | 34 |
| 3 kisses | 70 | 276 | 73 | 19 | 438 |
| sum | 191 | 321 | 77 | 24 | 613 |
