# Supplementary material for: Can Population-Level Laterality Stem from Social Pressures? Evidence from Cheek Kissing in Humans
Source: PLoS One. 2015 Aug 13;10(8):e0124477. doi: 10.1371/journal.pone.0124477 (PMC4536016; doi:10.1371/journal.pone.0124477)
Supplement: S4 Table — Correlogram showing the Moran’s intercorrelation index (I) according to distance. (DOC) [file pone.0124477.s004.doc]

**S4 Table. These are the raw data of Figure 7. Correlogram showing the Moran’s intercorrelation index (I) according to distance.**

|  |  | Class # | Min Dist | Max Dist | # Pairs | E(I) | I | SD(I) | Z(I) | Prob(I) | RandProb(I) |
| --- | --- | --- | --- | --- | --- | --- | --- | --- | --- | --- | --- |
| HI | Class 1 | 1 | 0 | 117634.4 | 291 | -0.01 | 0.37 | 0.06 | 6.81 | 0 | 0 |
| HI | Class 2 | 2 | 117634.4 | 173215.6 | 291 | -0.01 | 0.19 | 0.06 | 3.49 | 0 | 0 |
| HI | Class 3 | 3 | 173215.6 | 216198.9 | 292 | -0.01 | 0.17 | 0.06 | 3.26 | 0 | 0 |
| HI | Class 4 | 4 | 216198.9 | 254730.1 | 292 | -0.01 | 0.16 | 0.06 | 3.1 | 0 | 0 |
| HI | Class 5 | 5 | 254730.1 | 296961.7 | 290 | -0.01 | 0.23 | 0.06 | 4.3 | 0 | 0 |
| HI | Class 6 | 6 | 296961.7 | 331420.7 | 291 | -0.01 | 0.07 | 0.06 | 1.42 | 0.16 | 0.22 |
| HI | Class 7 | 7 | 331420.7 | 369400.5 | 293 | -0.01 | 0 | 0.06 | 0.24 | 0.81 | 0.96 |
| HI | Class 8 | 8 | 369400.5 | 400678.3 | 291 | -0.01 | -0.02 | 0.06 | 0.16 | 0.87 | 0.73 |
| HI | Class 9 | 9 | 400678.3 | 439052.1 | 291 | -0.01 | -0.11 | 0.06 | 1.68 | 0.09 | 0.07 |
| HI | Class 10 | 10 | 439052.1 | 477960.7 | 291 | -0.01 | -0.05 | 0.06 | 0.74 | 0.46 | 0.36 |
| HI | Class 11 | 11 | 477960.7 | 518194.3 | 292 | -0.01 | -0.05 | 0.06 | 0.73 | 0.46 | 0.36 |
| HI | Class 12 | 12 | 518194.3 | 564396 | 291 | -0.01 | -0.2 | 0.06 | 3.31 | 0 | 0 |
| HI | Class 13 | 13 | 564396 | 614592.2 | 291 | -0.01 | -0.21 | 0.06 | 3.53 | 0 | 0 |
| HI | Class 14 | 14 | 614592.2 | 685808.9 | 292 | -0.01 | -0.27 | 0.05 | 4.73 | 0 | 0 |
| HI | Class 15 | 15 | 685808.9 | 987393.6 | 292 | -0.01 | -0.45 | 0.05 | 8.59 | 0 | 0 |
